# Comprehensive evaluation of gene expression signatures in response to electroacupuncture stimulation at Zusanli (ST36) acupoint by transcriptomic analysis

**DOI:** 10.1186/s12906-017-1911-0

**Published:** 2017-08-15

**Authors:** Jing-Shan Wu, Hsin-Yi Lo, Chia-Cheng Li, Feng-Yuan Chen, Chien-Yun Hsiang, Tin-Yun Ho

**Affiliations:** 1 Graduate Institute of Chinese Medicine, China Medical University, 91 Hsueh-Shih Road, Taichung, 40402 Taiwan; 2 Department of Microbiology, China Medical University, 91 Hsueh-Shih Road, Taichung, 40402 Taiwan; 3 Department of Health and Nutrition Biotechnology, Asia University, Taichung, 41354 Taiwan

**Keywords:** Electroacupuncture, ST36, Zusanli, Microarray

## Abstract

**Background:**

Electroacupuncture (EA) has been applied to treat and prevent diseases for years. However, molecular events happened in both the acupunctured site and the internal organs after EA stimulation have not been clarified.

**Methods:**

Here we applied transcriptomic analysis to explore the gene expression signatures after EA stimulation. Mice were applied EA stimulation at ST36 for 15 min and nine tissues were collected three hours later for microarray analysis.

**Results:**

We found that EA affected the expression of genes not only in the acupunctured site but also in the internal organs. EA commonly affected biological networks involved in cytoskeleton and cell adhesion, and also regulated unique process networks in specific organs, such as γ-aminobutyric acid-ergic neurotransmission in brain and inflammation process in lung. In addition, EA affected the expression of genes related to various diseases, such as neurodegenerative diseases in brain and obstructive pulmonary diseases in lung.

**Conclusions:**

This report applied, for the first time, a global comprehensive genome-wide approach to analyze the gene expression profiling of acupunctured site and internal organs after EA stimulation. The connection between gene expression signatures, biological processes, and diseases might provide a basis for prediction and explanation on the therapeutic potentials of acupuncture in organs.

## Background

Acupuncture, a traditional therapy in ancient China over thousands of years, has been widely accepted and used in Western society [1, 2]. Acupuncture is believed to balance Yin-Yang, stimulate the circulation of vital energy (qi) and blood, maintain the body health, and prevent the incidence of illness [3]. Electroacupuncture (EA) is a modification of acupuncture that stimulates acupoints with electrical current and displays reproducible in both research and clinical application. Moreover, EA therapy has been used for postoperative analgesia and anesthesia, for the treatment of diverse disorders of internal organs, and for the release of pain [4, 5].

In traditional Chinese medicine, ST36 (Zusanli) is a commonly acupoint that modulates the biological activities of gastrointestinal system, immune system, cardiovascular system, and muscular system. Transcutaneous EA at ST36 reduces gastric accommodation and improves impaired gastric motility in patients with functional dyspepsia [6]. Transcutaneous neuromodulation at ST36 also improves the frequency of spontaneous defecation and increases the bowel movements in patients with chronic constipation [7]. Chronic EA at ST36 improves baroreflex function and hemodynamic parameters in rats with heart failure [8]. Long-term EA stimulation at ST36 and DU20 (BaiHui) also relieves the increased mean arterial pressure and cardiovascular abnormality in both structure and function in spontaneously hypertensive rats [9]. ST36 displays the anti-nociceptive and anti-hyperalgesic effect. EA at ST36 reduces postoperative analgesic requirements and associated side effects in patients undergoing lower abdominal surgery [10]. Treatment of EA at ST36 and GN34 also ameliorates L5 spinal nerve ligation-induced neuropathic pain in rats [11]. Moreover, EA at ST36 and CV4 (Guanyuan) improves clinical curative effects in patients with sepsis in a prospective randomized controlled trial via the regulation of immune system [12]. EA at ST36 promotes myofiber regeneration and restoration of neuromuscular junctions in a rabbit gastrocnemius contusion model [13]. Furthermore, EA at ST36 also improves intestinal mucosal immune barrier in sepsis by increasing the concentration of secretory IgA, the percentage of CD3+, γ/δ, and CD4+ T cells, and the ratio of CD4+/CD8+ T cells [14].

ST36 is an acupoint of Foot’s Yang Supreme Stomach Meridian that targets at gastrointestinal tract [15]. We wondered whether EA stimulation at ST36 altered molecular events in other organs. The genome-wide analysis of ST36-stimulated region (skin) and distant visceral organs or tissues, including cerebral medulla, cerebral cortex, hippocampus, lung, spleen, kidney, uterus and thigh muscle, was therefore performed. Mice were stimulated by EA at ST36, and gene expression signatures of nine organs or tissues were explored by microarray analysis. The process network and disease connection of gene expression profiles were further analyzed to elucidate the molecular events and effects of organs after ST36 stimulation.

## Methods

### Animals

Female BALB/c mice (6–8-week-old, 18–22 g) were obtained from National Laboratory Animal Center (Taipei, Taiwan) and maintained in an air-controlled pathogen-free animal facility with a 12-h light/dark cycle at 23 ± 2 °C. Food and water were available ad libitum. Mouse experiments were conducted under ethics approval from China Medical University Animal Care and Use Committee (Permit No. 101–61-N).

### EA stimulation

A total of 10 mice was randomly divided into two groups of 5 mice. For control group, mice were anesthetized with isoflurane without ST36 stimulation. For ST36 group, mice were anesthetized with isoflurane, gently immobilized in a plastic restrainer, and applied EA stimulation at ST36 acupoint, which is located at the midpoint of tibialis anterior muscle of hind limbs. Briefly, sterilized acupuncture needle (0.24 × 12 mm, 36 gauge, Yu-Kuang Acupuncture Co., Taipei, Taiwan) was inserted bilaterally into the acupoint, which was 3–4 mm below the knee midline and laterally 1–2 mm at a depth of 2–3 mm. Electrical stimulation pulse with voltage ranging from 3.5 to 5 V, duration of 0.05 ms, and frequency of 2 Hz was generated from a pulse generator (HANS model, LH202H, Taipei, Taiwan) and applied using two outlets via two needles. The intensity of EA stimulation was determined as the minimum voltage causing moderate muscle contraction for 15 min. Three hours after EA stimulation, mice were sacrificed by carbon dioxide inhalation, and organs were removed for RNA extraction.

### Total RNA extraction

Total RNAs from acupunctured site, muscle, cerebral cortex, cerebral medulla, hippocampus, lung, spleen, kidney, and uterus were extracted using RNeasy Mini kit (Qiagen, Valencia, CA, USA). The amount and the integrity of total RNA were quantified and evaluated using a spectrophotometer (Beckman Coulter, Fullerton, CA, USA) and an Agilent 2100 bioanalyzer (Agilent Technologies, Santa Clara, CA, USA), respectively.

### Microarray analysis

Microarray analysis was performed as described previously [16, 17]. Briefly, fluorescence-labeled RNA targets were prepared from total RNA using MessageAmp™ aRNA kit (Ambion, Austin, TX, USA) and Cy5 dye (Amersham Pharmacia, Piscataway, NJ, USA). Fluorescent targets were hybridized to the Mouse Whole Genome OneArray (Phalanx Biotech Group, Hsinchu, Taiwan) and scanned by an Axon 4000 scanner (Molecular Devices, Sunnyvale, CA, USA). The Cy5 fluorescent intensity of each spot was analyzed by genepix 4.1 software (Molecular Devices, Sunnyvale, CA, USA). The signal intensity of each spot was normalized by R program in limma package using quantile normalization. Normalized data were analyzed using the “geneSetTest” function implemented in the limma package to detect groups of regulated genes in biological pathways. This function computes a *p*-value to test the hypothesis that the selected genes tend to be up- or down-regulated. Then, the score of each pathway in EA treatment was defined as score = − log(*p*) if *p*-value ≤0.5 or score = log(2(1 − *p*)) if *p*-value >0.5. The score more than 0.3, equivalent to *p*-value less than 0.5, was considered to be statistically significant. A total of 352 pathways was extracted from ArrayTrack (http://www.fda.gov/ScienceResearch/BioinformaticsTools/Arraytrack) and used in this analysis. The scores of pathways were then displayed using TIGR Multiexperiment Viewer (http://mev.tm4.org) [18]. In addition to biological pathways analysis, genes with fold changes ≥1.5 or ≤ −1.5 were selected and used as input genes for the generation of process network and diseases using Enrichment algorithm in MetaCore™ Analytical suit (GeneGo Inc., St. Joseph, MI, USA). All microarray data are MIAMI compliant database (Gene Expression Omnibus accession number GSE73939).

## Results

### EA affected the expression of genes in distant organs

**Fig. 1:**
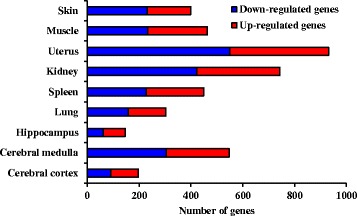
Number of ST36-regulated genes in various organs. EA stimulation was applied at ST36 acupoint in mice for 15 min. Three hours later, mice were sacrificed and organs were collected for microarray analysis. Data are presented by histograms, and the height of histogram corresponds to the number of upregulated (*red*) and downregulated (*blue*) genes

To explore the molecular events happened in local or distant regions after ST36 stimlation, we applied EA stimulation in BALB/c mice for 15 min and collected ST36-stimulated region (skin) and distant visceral organs or tissues, including cerebral medulla, cerebral cortex, hippocampus, lung, spleen, kidney, uterus and thigh muscle, 3 h later for microarray analysis. BALB/c mice were applied for EA stimulation in this study because BALB/c mice are among the most widely used inbred strains for animal experiments. Moreover, BALB/c mice are useful for researches of immunology and neurobiology, the potent biological activities of ST36 acupoint. As expected, EA affected the expression of genes in the skin at ST36 acupoint (Fig. 1). In a total of 29,922 genes, the transcripts of 169 genes and 231 genes were upregulated and downregulated, respectively, by 1.5 fold in EA-treated skin. In addition to skin, EA affected the expression levels of genes in distant organs. EA regulated the expression of 931 genes in uterus, followed by kidney (743 genes), cerebral medulla (547 genes), muscle (463 genes), spleen (450 genes), lung (303 genes), cerebral cortex (197 genes), and hippocampus (147 genes).

### EA affected biological processes in various organs

**Fig. 2:**
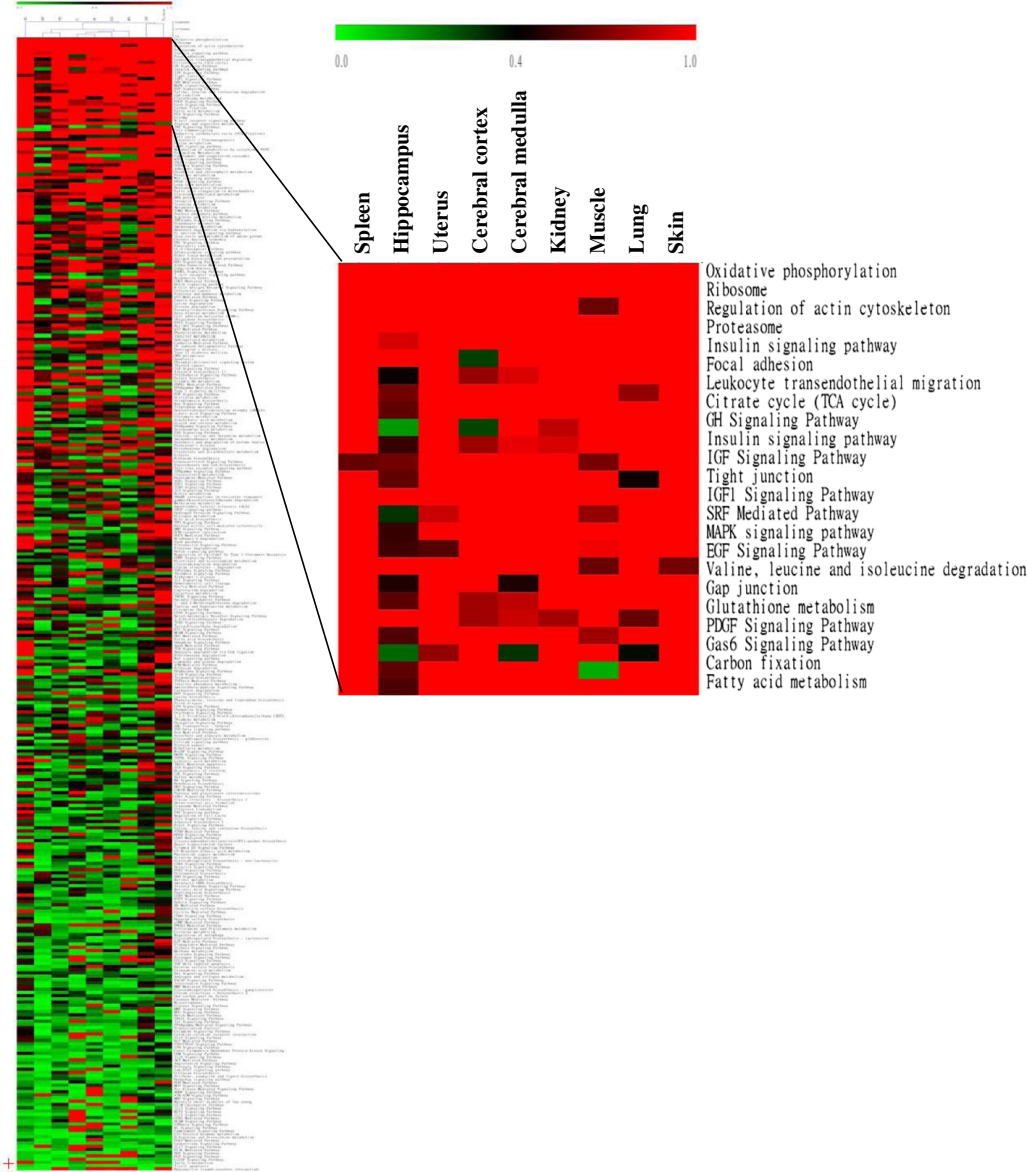
Hierarchical clustering analysis of biological pathways regulated by EA stimulation at ST36 acupoint in various organs. The score of each pathway is color-coded according to the legend at the *top*. Top 25 pathways are enlarged and shown on the *right panel*

We further analyzed the canonical pathways affected by EA at ST36 acupoint. “geneSetTest” function was performed to test a set of signaling and metabolic pathways regulated by EA. Scores of pathways were further visualized by TIGR Multiexperiment Viewer. As shown in Fig. 2, a hierarchical clustering of EA-affected canonical pathways displayed varieties among nine organs or tissues, and the number of signaling and metabolic pathways significantly regulated (score ≥ 0.3) by EA in different organs was also varied. Some pathways, such as oxidative phosphorylation, ribosome, proteasome and serum response factor-mediated pathways, were commonly regulated by EA in organs. However, more pathways were regulated by EA in organs without consistency. About 2/3 pathways in spleen and skin were significantly regulated by EA, while less pathways were regulated by EA in hippocampus.

**Fig. 3:**
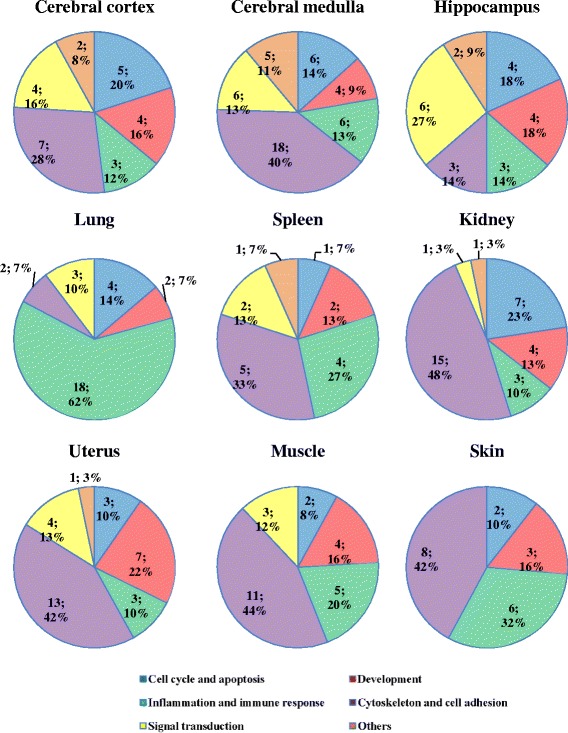
Process network categories affected by EA stimulation at ST36 acupoint in various organs. Genes with fold changes ≥1.5 or ≤ −1.5 were selected for the generation of process networks using MetaCore Analytical suit. Process networks were sorted into five categories and the categories were illustrated at the *bottom*. The pie chart sectors represent the number and the ratio of process networks in each category

**Fig. 4:**
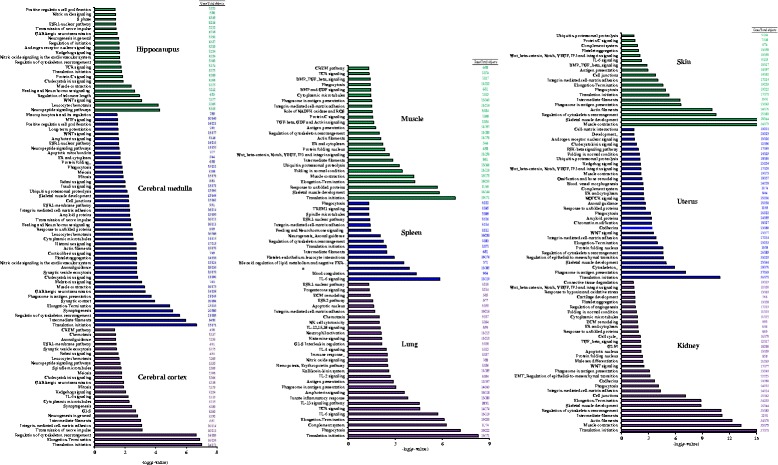
List of process networks regulated by EA stimulation at ST36 acupoint in various organs. Genes with fold changes ≥1.5 or ≤ −1.5 were selected for the generation of process networks using MetaCore Analytical suit. Data are presented as -log(*p*-value). The number of affected genes and total objects in each process network is shown on the *right panel*

We further analyzed the process network of protein interactions regulated by EA treatment and classified the process networks into five categories, including cell cycle and apoptosis, inflammation and immune response, signaling transduction, cytoskeleton and cell adhesion, and development. EA treatment affected the process networks in these organs in different ratio (Fig. 3). Cytoskeleton and cell adhesion was the most EA-regulated category in organs, except hippocampus and lung. “Inflammation and immune response” was the most affected category in lung. About 60% of the total number of EA-affected process networks was related to inflammation and immune response. Signaling transduction was the most affected category in hippocampus, and approximately 27% of the total number of affected process networks was involved in signaling transduction. In addition, EA treatment affected some unique process networks in organs (Fig. 4). For example, some neurophysiological processes, such as transmission of nerve impulse and γ-aminobutyric acid-ergic (GABAergic) neurotransmission, were commonly regulated by EA in cerebral cortex, cerebral medulla, and hippocampus, while melatonin signaling, corticoliberin signaling, and long-term potentiation were regulated by EA in cerebral medulla. Moreover, male sex differentiation in kidney, follicle-stimulating hormone-beta signaling pathway in uterus, and blood coagulation in spleens were significantly affected by EA.

### Gene expression connection between EA stimulation and diseases in brain and lung

EA stimulation at ST36 regulated the expression of about 300–500 genes in lung and brain. Although the organs with the top two changes in gene expression were uterus and kidney, the ratios of process network categories altered by uterus and kidney were similar to those altered by other organs, except brain and lung. Process network analysis showed that “inflammation and immune response” was the abundant category in lung and neurological processes were unique processes regulated in brain. Therefore, we further analyzed whether genes affected by EA were related to those in diseases. As shown in Table 1, EA stimulation commonly regulated the genes involved in psychiatry and psychology, mental disorders, mood disorders, and heredodegenerative disorders in brain tissues. EA treatment also regulated the expression of genes related to some unique diseases in brain tissues. For example, genes involved in neurodegenerative diseases, such as Alzheimer’s disease, amyloid neuropathies and parkinsonian disorders, were regulated by EA in cerebral medulla. Genes related to endocrine system diseases, such as ovarian diseases, adnexal diseases, ovarian neoplasms, gonadal disorders, and prostatic intraepithelial neoplasia, were regulated by EA in hippocampus. As shown in Table 2, genes involved in obstructive pulmonary diseases, hypersensitivity, such as rheumatic diseases and rheumatoid arthritis, infection, such as bacterial infections and mycoses, and cardiopulmonary diseases, such as cardiovascular diseases, heart diseases and vascular diseases, were affected by EA in lung. These findings suggested that EA stimulation at ST36 acupoint affected the biological process and network in distant organs. Moreover, EA-affected gene expression profiles might be related to diseases states in brain and lung.

**Table 1:** Top 20 diseases affected by EA stimulation at ST36 in brain tissues

Diseases	*p*-value	Gene/Total objects
Cerebral cortex		
Psychiatry and Psychology	4.95E-16	55/1875
Dementia	6.28E-15	47/1480
Central Nervous System Diseases	7.87E-13	65/2983
Mental Disorders	1.65E-12	45/1593
Brain Diseases	1.79E-12	62/2804
Depressive Disorder	3.92E-12	26/557
Neurodegenerative Diseases	1.14E-11	50/2030
Delirium, Dementia, Amnestic, Cognitive Disorders	1.73E-11	15/164
Chorea	2.58E-11	23/467
Dyskinesias	6.16E-11	28/733
Huntington Disease	1.21E-10	22/459
Diabetes Insipidus, Neurogenic	2.95E-10	5/6
Movement Disorders	4.92E-10	29/859
Nervous System Diseases	7.33E-10	86/5368
Mood Disorders	1.55E-09	27/789
Hyponatremia	2.71E-09	42,498
Intellectual Disability	4.60E-09	22/558
Basal Ganglia Diseases	7.57E-09	26/792
Heredodegenerative Disorders, Nervous System	1.03E-08	30/1045
Behavior and Behavior Mechanisms	1.11E-08	20/484
Cerebral medulla		
Mental Disorders	1.15E-32	124/1593
Psychiatry and Psychology	3.08E-32	135/1875
Schizophrenia and Disorders with Psychotic Features	1.99E-29	88/914
Neurodegenerative Diseases	1.50E-19	117/2030
Basal Ganglia Diseases	1.50E-17	64/792
Brain Diseases	3.66E-17	138/2804
Dementia	7.17E-17	91/1480
Movement Disorders	2.13E-16	65/859
Central Nervous System Diseases	1.08E-15	140/2983
Amyloid Neuropathies	3.68E-15	42,655
Tauopathies	1.57E-14	72/1113
Heredodegenerative Disorders, Nervous System	2.17E-13	67/1045
Mood Disorders	1.45E-12	55/789
Neurologic Manifestations	6.85E-12	81/1511
Pathological Conditions, Signs and Symptoms	8.83E-12	169/4334
Alzheimer Disease	1.86E-11	65/110
Parkinsonian Disorders	5.34E-11	35/401
Cerebral Hemorrhage	1.36E-10	15/72
Alzheimer disease, early onset	2.51E-10	12/43
Lewy Body Disease	7.83E-10	12/43
Hippocampus		
Mental Disorders	4.44E-07	27/1593
Psychiatry and Psychology	1.01E-06	29/1875
Schizophrenia and Disorders with Psychotic Features	8.91E-05	16/914
Ovarian Diseases	1.19E-04	34/3046
Adnexal Diseases	1.21E-04	34/3049
Ovarian Neoplasms	1.28E-04	33/2927
Behavioral Symptoms	1.91E-04	6/142
Osteoarthritis, Knee	1.93E-04	4/49
Gonadal Disorders	1.99E-04	34/3128
Behavior	2.76E-04	6/152
Dyskinesias	3.73E-04	13/733
Huntington Disease	3.77E-04	10/459
Prostatic Intraepithelial Neoplasia	3.87E-04	7/227
Heredodegenerative Disorders, Nervous System	4.05E-04	16/104
Chorea	4.31E-04	10/467
Craniofacial Abnormalities	4.64E-04	7/234
Suicide	5.83E-04	5/115
Self-Injurious Behavior	5.83E-04	5/115
Mood Disorders	7.43E-04	13/789
Endocrine System Diseases	9.26E-04	50/5714

**Table 2:** Top 20 diseases affected by EA stimulation at ST36 in lung

Diseases	*p*-value	Gene/Total objects
Pathologic Processes	7.75E-28	101/2642
Pulmonary Disease, Chronic Obstructive	8.21E-26	57/881
Nutritional and Metabolic Diseases	8.30E-25	105/3099
Metabolic Diseases	4.00E-23	96/2765
Pathological Conditions, Signs and Symptoms	6.83E-23	123/4334
Connective Tissue Diseases	3.75E-21	82/2213
Rheumatic Diseases	2.30E-20	67/1563
Lung Diseases, Obstructive	6.69E-20	68/1640
Bacterial Infections and Mycoses	7.73E-20	60/1293
Arthritis	9.28E-20	68/1650
Arthritis, Rheumatoid	1.83E-19	63/1446
Joint Diseases	2.43E-19	68/1680
Infection	2.11E-18	55/1171
Hypersensitivity	3.60E-18	63/1535
Hypersensitivity, Immediate	4.38E-18	59/1362
Wounds and Injuries	6.15E-18	50/995
Cardiovascular Diseases	8.12E-18	100/3520
Heart Diseases	2.58E-16	56/1351
Vascular Diseases	3.33E-16	91/3179
Fibrosis	5.08E-16	33/475

## Discussion

In this study, we applied transcriptomic analysis to analyze the gene expression signatures in nine organs or tissues responsive to ST36 stimulation. Microarray analysis has been applied to elucidate the effects of various acupoints in specific organs or tissues. For example, acupuncture at GB34 and LR3 acupoints attenuates the decrease of tyrosine hydroxylase and exhibits the protective effects via affecting the expression of degeneration-related genes in the substantia nigra region in Parkinsonism mouse model [19]. Acupuncture at PC6 acupoint up-regulates the expression of Tph1 gene and down-regulates the expression of Olr883 genes in rat brains, suggesting that the therapeutic effect of acupuncture for ischemic stroke may be closely related to the suppression of post-stroke depression and the regulation of olfactory transduction in middle cerebral artery occlusion rat model [20]. Moreover, EA at PC3 and PC6 acupoints significantly ameliorates the colonic lesions, and affects both the inflammatory pathways in colons and the immunity-associated pathway in spleens in mice with trinitrobenzene sulfuric acid- induced colitis [21]. Gene expression profiles of specific organs or tissues after EA stimulation at ST36 have also been analyzed. For example, gene expression profiles in periaqueductal gray-spinal dorsal horn region of rats after EA stimulation at ST36 and SP6 show that the modulation of neural-immune interaction in the central nervous system plays an important role during EA analgesia [22]. Gene expression profiling of rat arcuate nucleus region responsive to EA at ST36 and SP6 shows that the expression levels of genes are effectively regulated by low-frequency EA, compared with high-frequency EA. It might explain the mechanisms of therapeutic effects of the low-frequency EA [23]. In addition to brain tissues, EA at ST36 affects the expression of cell adhesion molecules in muscle, which might be related to the glucose-lowering effect of ST36 in rats with type 1 diabetes [24]. Acupuncture at ST36, CV12 (Zhongwan), and BL20 (Pishu) acupoints down-regulates nuclear factor-κB p65, miRNA-155, and miRNA-21 and up-regulates miRNA-146a expression in chronic atrophic gastritis rats, suggesting that these genes may play important roles in therapeutic effect of acupuncture in treating chronic atrophic gastritis [25]. Moreover, moxibustion at ST36 affects the biological processes involved in immunity and metabolism in moxibustioned skin under pathological and physiological conditions, respectively [26]. Since ST36 displays various benefit or therapeutic effects in whole bodies, we performed a global and comprehensive study on the gene expression signatures of nine different organs or tissues after ST36 stimulation. Our data showed that EA at ST36 affected the gene expression of different organs or tissues in various degrees. Moreover, EA at ST36 has a more impact on the regulation of gene expression in uterus and has a lesser impact in hippocampus.

By process network and disease connection analysis, we found that EA at ST36 affected the process networks involved in inflammation and immune responses in lung and affected the expression of genes involved in respiratory diseases, such as obstructive pulmonary diseases and microbial infection. Interestingly, a prospective single-blind randomized placebo-controlled study shows that transcutaneous electrical nerve stimulation at ST36, EX-B-1(Dingchuan), BL13 (Feishu), and BL23 (Shenshu) improves lung function on patients with stable chronic obstructive pulmonary disease [27]. Another study shows that EA at ST36 and BL13 improves lung function of rats with chronic obstructive pulmonary disease and displays an anti-inflammatory effect via downregulation of orexin and its receptor [28]. In addition, EA at ST36 displays a potential protective effect on severe thermal injury-induced remote acute lung injury via the limitation of inflammatory responses in rats [29]. Moreover, EA treatment at ST36 and BL13 attenuates lung injury in rats with endotoxic shock-induced acute lung injury through the activation of NF-E2-related factor pathway and the up-regulation of heme oxygenase-1 expression [30]. Acupuncture at ST36 also regulates the disorders of Fas and Bcl-2 mRNA expression, promotes the apoptosis of eosinophils, and consequently inhibits the development of inflammatory reaction of asthma in rats [31].

Acupuncture has shown some benefit effects on Alzheimer’s disease and Parkinson’s disease. Lu et al. [32] showed that acupuncture at ST36 increases blood perfusion and glycol metabolism in certain brain areas in Alzheimer’s disease rat model by Positron Emission Tomography scanning. ST36 stimulation also induces neurogenesis in adult brains via the up-regulations of brain-derived neurotrophic factor, glial cell line-derived neurotrophic factor, basic fibroblast growth factor and neuropeptide Y, and the activation of the function of primo vascular system [33]. By database searching and screening for articles on clinical trials, Feng et al. [34] found that ST36 combined with GV20 (Baihui) or GV24 (Shenting) is the most frequent and represent potential combination for vascular dementia treatment. In addition, acupuncture at ST36 improves cognitive deficits and increases pyramidal neuron number of hippocampal CA1 area in vascular dementia rats [35]. Moreover, EA at ST36 alleviates dementia via the modulation of interneuron function and the increases of long-term potentiation of hippocampus in rats [36]. By analyzing the gene expression profiling of cerebral cortex, cerebral medulla, and hippocampus after ST36 stimulation, we found that stimulation at ST36 affected the expression of genes involved in neurodegenerative diseases, such as Alzheimer’s disease and Parkinsonian disorder, and mental disorders, such as dementia. In addition, neurophysiological processes, such as GABAergic neurotransmission and long-term potentiation, were also regulated by ST36 stimulation in brains. The connection between gene expression signatures in brain and neurological diseases might provide an explanation on the therapeutic effects of acupuncture for neurological diseases.

In this study, we found that, in addition to brain and lung, EA stimulation at ST36 affected the expression of genes in the local region, such as acupunctured skin, and in the distant regions, like muscle, uterus, kidney, and spleen. How can the stimulation at body surface affect the gene expression in the internal region far from the acupunctured site? Autonomic nervous system is frequently considered to be a mediator of acupuncture. Vagus nerve is a primary target for exploring the possible effect of acupuncture on internal organs because vagus nerve broadly regulates the functions of internal organs. Acupuncture stimulation raises the vagal tone and consequently affects the heart rate and the arterial pressure of cardiovascular system, and the intestinal motility of gastrointestinal system [37]. Acupuncture also exhibits anti-inflammatory effects via vagal modulation of inflammatory responses in internal organs. For example, acupuncture at ST36 activates the splenic nerve via vagus nerve activity to induce anti-inflammatory responses in macrophages of spleens in a lipopolysaccharide-induced inflammation rat model [3]. EA also controls systemic inflammation by inducing vagal activation of aromatic L-amino acid decarboxylase, leading to the production of dopamine in the adrenal medulla and the inhibition of cytokine production [38]. Some neurotransmitters are involved in the transmission of acupuncture stimulation to nerves. Tjen-A-Looi et al. [39] showed that EA at P5 and P6 acupoints restores the blood pressure in phenylbiguanide-induced hypotension and bradycardia cat models through both opioid and GABAergic processing mechanisms. They also showed that EA at P5 and P6 modulates the cardiovascular depressor responses during gastric distention in rats via GABAergic mechanisms [40]. Our data also showed that gene expression signatures responsive to ST36 stimulation connected to the GABAergic neurotransmission network in brain.

## Conclusions

In conclusion, we performed a global comprehensive study on the gene expression signatures of nine different organs or tissues after ST36 stimulation. EA at ST36 affected the expression of genes not only in acupunctured site but also in internal organs. Gene expression signatures showed that stimulation at ST36 acupoint commonly affected process networks involved in cytoskeleton and cell adhesion in these organs. However, EA at ST36 also regulated unique process networks in specific organs or tissues. In addition, ST36 stimulation affected the expression of genes related to various diseases. The connection between gene expression signatures and diseases might provide a basis for the prediction and the explanation on the therapeutic potentials of acupuncture in various organs.

## Abbreviations

EA: Electroacupuncture
GABAergic: γ-aminobutyric acid-ergic
